# Demonstration of reduced efficacy against cyathostomins without change in species composition after pyrantel embonate treatment in Swedish equine establishments

**DOI:** 10.1016/j.ijpddr.2023.11.003

**Published:** 2023-11-14

**Authors:** Ylva Hedberg Alm, Peter Halvarsson, Frida Martin, Eva Osterman-Lind, Vendela Törngren, Eva Tydén

**Affiliations:** a Department of Biomedical Science and Veterinary Public Health, Parasitology Unit, Swedish University of Agricultural Sciences, SE-750 07, Uppsala, Sweden; b Department of Microbiology, Section for Parasitology, National Veterinary Institute (SVA), SE-751 89, Uppsala, Sweden

**Keywords:** Anthelmintic resistance, Cyathostomins, FECRT, Metabarcoding, Pyrantel, Small strongyles

## Abstract

Consisting of approximately 50 different species, the cyathostomin parasites are ubiquitous in grazing horses. Co-infection with several species is common, and large burdens can cause the fatal disease of larval cyathostominosis. Due to intense anthelmintic drug use, cyathostomin resistance has developed to all available anthelmintic drug groups. Resistance to the anthelmintic drug pyrantel (PYR) has been documented in over 90% of studies published over the past two decades. In Sweden, a study performed in the early 2000s only confirmed resistance in 4.5% of farms. Further, prescription-only administration of equine anthelmintic drugs was enforced in Sweden in 2007. However, it is unknown if this conservative drug use has maintained PYR efficacy in cyathostomins. The aim of the present study was to investigate the effect of PYR on cyathostomin infection in Sweden using fecal egg count reduction tests (FECRTs). Further, the effect of PYR treatment on cyathostomin species composition was studied using metabarcoding.

Sixteen farms with at least six horses excreting a minimum of 100 eggs per gram feces were included. Using the current World Association for the Advancement of Veterinary Parasitology (WAAVP) guidelines, PYR resistance was demonstrated in nine of farms, with seven farms showing full susceptibility. Farms with low biosecurity measures had significantly lower efficacy of PYR treatment. The most common cyathostomin species were *Cylicocyclus nassatus, Cyathostomum catinatum, Cylicostephanus longibursatus, Cys. calicatus, Cys. goldi, Cys. minutus, Coronocyclus coronatus* and *Cya. pateratum*, accounting for 97% of all sequence reads prior to treatment. Of these, *Cyc. nassatus* and *Cya. catinatum* had the highest occurrence, accounting for 68% of all sequence reads prior to PYR treatment. Treatment did not significantly affect the species composition. The results highlight the importance of drug efficacy testing when using PYR to treat cyathostomin infection, even when selective anthelmintic treatment and thus low treatment intensity, is used on the farm.

## Highlights

• Pyrantel resistance in cyathostomins despite selective anthelmintic strategy.
• Poor biosecurity measures associated with lower pyrantel drug efficacy.
• Efficacy testing of pyrantel vital, regardless anthelmintic treatment regime.
• No change in cyathostomin species composition after pyrantel treatment.

## Graphical abstract

**Figure:**
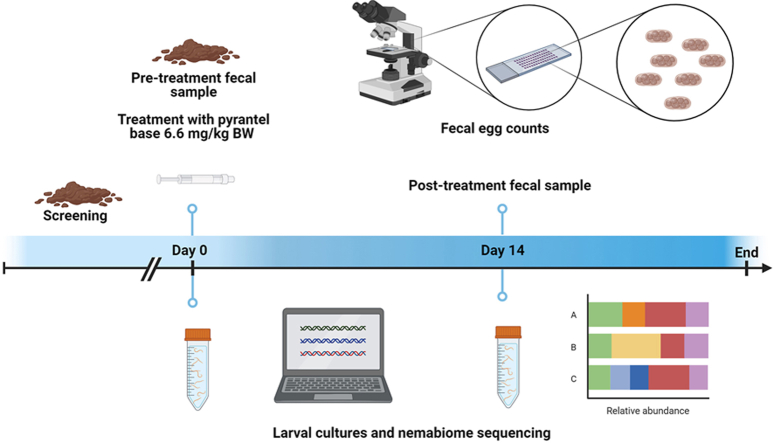

## 1 Introduction

Cyathostominae or small strongyles are the most abundant equine internal gastrointestinal parasites and are considered ubiquitous in horses worldwide. Around 50 different species of cyathostomins have been described and an individual horse may be co-infected with as many as 15–25 species (Reinemeyer et al., 1984; Chapman et al., 2003; Bellaw and Nielsen, 2020). Although not as pathogenic as *Strongylus vulgaris*, one of the large strongyles, there are indications that large burdens of cyathostomins can cause loss of weight and body condition (Love et al., 1999; Murphy and Love, 1997; Reinemeyer et al., 2014). In addition, young horses are susceptible to a serious and sometimes fatal condition known as larval cyathostominosis, which can occur when large numbers of encysted larvae simultaneously emerge from the large intestinal mucosa (Love et al., 1999; Peregrine et al., 2006; Lawson and Mair, 2023).

The anthelmintic drug classes available for treatment of equine nematodes are confined to three different drug classes, namely benzimidazoles (BZ), tetrahydropyrimidines (THP) and macrocyclic lactones (ML). Unfortunately, parasite control based on year-round interval treatment for several decades has led to the emergence of drug resistance in cyathostomins to all three drug classes. There is a global widespread resistance to BZ and this drug class can no longer be recommended for the treatment of cyathostomins (Kaplan, 2002; Nielsen et al., 2014a; Nielsen, 2022). Furthermore, there is increasing evidence of resistance of cyathostomins to THP and more recently, several reports documenting a lack of efficacy to MLs, with confirmed resistance in cyathostomins to MLs published in 2020 (Nielsen et al., 2020; Nielsen, 2022). Unfortunately, there is no indication of any new classes of equine anthelmintic drugs becoming available in the foreseeable future (Nielsen, 2022), and therefore, selective anthelmintic treatment, a strategy suggested already in the early 1990s, should be enforced (Duncan and Love, 1991; Gomez and Georgi, 1991). By treating only those individuals which excrete above a set threshold of parasite eggs, often 200 eggs per gram feces (EPG), the usage of anthelmintic drugs is reduced, with the aim to slow down further development of resistance (Nielsen et al., 2019a, Nielsen et al., 2019b; Geurden et al., 2021). In addition, due to the dynamic evolvement of resistance over time and the risk of introduction of resistant parasite species with the movement of new horses onto equine premises (Nielsen et al., 2020), regular monitoring of the efficacy of the anthelmintic drugs in use is vital (Nielsen, 2021). The most commonly used *in vivo* test for anthelmintic resistance (AR) in horses is the fecal egg count reduction test (FECRT), where pre-treatment egg excretion is compared with egg excretion 14 days post-treatment (Kaplan et al., 2023). In order to elucidate whether there are species differences in anthelmintic drug efficacy and resistance development, the method of metabarcoding, that is, using barcoding of ribosomal DNA (rDNA) to determine species composition, has recently been employed as regards to MLs (Nielsen et al., 2022). Metabarcoding was also recently used to describe the cyathostomin species population prior to and after PYR treatment, although the primary aim of the study was to investigate the interaction between cyathostomins and the gut microbiota (Boisseau et al., 2022).

In Sweden, ML is the most widely used anthelmintic drug class for the treatment of equine strongyle infections (Tydén et al., 2019; Hedberg-Alm et al., 2020). However, sole reliance on a single drug class group may be unadvisable, given that lowered efficacy to MLs, although initially slow in development, is now being documented in several countries (Nielsen, 2022). Pyrantel (PYR), an anthelmintic drug belonging to the THPs, could be an alternative drug for control of cyathostomin infection, if retained efficacy is confirmed. Early data for PYR showed an expected efficacy against susceptible cyathostomins of 96–100%, albeit with considerable variation both within and between horse farms (Lyons et al., 1975; Drudge et al., 1982). Since the year 2000, 37 studies investigating the presence of cyathostomin resistance to the THPs have been published, with resistance documented in 92% of them, including studies performed in Brazil, the USA, Morocco, Australia, New Zealand and various European countries (Nielsen, 2022). Only two of the countries (Turkey and South Africa) where PYR efficacy was studied did not confirm PYR resistance in cyathostomins (Davies and Schwalbach, 2000; Cirak et al., 2004). The most recent report evaluating the efficacy of PYR in treating cyathostomin infections in horses in Sweden was published in 2007 (Osterman Lind et al., 2007). In this study, resistance was initially suspected in six out of 22 farms investigated, and subsequently confirmed by repeated testing in one equine establishment, with the conclusion that PYR at that time could be considered a viable option for treatment of cyathostomin infections in Sweden. However, the current efficacy status of PYR in Sweden is unknown.

The primary aim of the present study was to evaluate the efficacy of a single oral dose of PYR in Swedish equine establishments using the FECRT, with metabarcoding employed to evaluate the effect of PYR treatment on cyathostomin species composition. In addition, a secondary aim was to investigate the influence of demographics, pasture management practices and anthelmintic routines on drug efficacy.

## 2 Materials and methods

### 2.1 Study design

Equestrian establishments were identified and recruited to the study through the National Veterinary Institute's (SVA) equine parasite monitoring program in April and May 2022, and establishments with a minimum of six horses excreting at least 100 eggs per gram feces (EPG) at day 0, prior anthelmintic treatment, were considered eligible for inclusion in the study. The included horses had not received anthelmintic treatment within the past six months. Fecal samples, obtained by either the horse owner or other responsible personnel at the establishment, were taken prior to and 14 days after treatment with a single oral dose of 6.6 mg pyrantel base/kg bodyweight (Banminth® pyrantel embonate 439 mg/g, Zoetis Animal Health ApS, Stockholm, Sweden). The weight of each horse was estimated using a weight tape (Boehringer Ingelheim AB, Stockholm, Sweden) and rounded up to the nearest 50 kg to minimize the risk of administrating an inadequate dose of anthelmintic drug.

### 2.2 Fecal analysis and larval cultures

Strongyle fecal egg counts (FECs) were determined for each horse using a modified centrifugation-enhanced McMaster technique using a multiplication factor of 12.5 (Coles et al., 1992). Nematode eggs in fecal samples (6 g) were flotated using a saturated NaCl solution with a density of SG = 1.18 g/cm^3^ (Coles et al., 1992). All samples were performed in duplicates. Larval cultures were performed on 20 g feces from each horse according to Bellaw and Nielsen (2015) irrespective of FEC. In brief, feces were mixed with an equal volume of vermiculite (Weibulls, Econova, Åby, Sweden) (Bellaw and Nielsen, 2015). Tap water was added to obtain a moist condition and samples were cultured at +20 °C for 14 d. Third stage larvae (L3) were harvested after sedimentation for 12–16 h at +20 °C by the inverted Petri dish method (van Wyk and Mayhew, 2013). To collect L3, approximately 20 ml of the fluid was collected into a 50 ml Falcon tube, and centrifuged at 248×*g* for 3 min. The supernatant was discarded leaving pelleted L3.

### 2.3 Calculation of fecal egg count reduction and definition of anthelmintic resistance

Mean FECs and mean fecal egg count reductions (FECRs) with corresponding credible intervals for each establishment before treatment (FECs only) and at 14 days post-treatment were calculated using a hybrid Frequentist/Bayesian analysis method (http://www.fecrt.com) (Denwood et al., 2019; Denwood et al., 2023). Anthelmintic resistance (AR) to PYR was concluded if the upper 90% confidence level (UCL) was less than an expected efficacy of 98%, according to the currently accepted definition sanctioned by the WAAVP (Kaplan et al., 2023). The guideline's research protocol was used and full susceptibility to PYR was deduced if the lower 90% confidence level (LCL) was ≥88% and the UCL ≥98%, with results showing an UCL ≥98%, but a LCL <88%, considered inconclusive (Kaplan et al., 2023).

### 2.4 Molecular methods

For each individual sample (before and after treatment for each horse, n = 264), DNA was extracted from approximately 2000 larvae using NuceloSpin®Tissue (Macherey-Nagel, Germany) according to the manufacturer's instruction. The concentration and quality of the DNA were analyzed using nanodrop (Nanodrop ND-1000, Thermo Fisher Scientific, Waltham, USA). Each sample was amplified using the universal nematode internal transcribed spacer region 2 (ITS2) ribosomal DNA primers (NC1-NC2) (Gasser et al., 1993), which were combined with unique 8-bp barcodes prior to pooling of the samples for sequencing (Ihrmark et al., 2012). Polymerase chain reactions (PCRs) were performed in duplicated 50-μl reactions using DreamTaq Green PCR Master Mix (2X) (Thermo Fisher Scientific, Waltham, USA) with a final primer concentration of 0.64 mM, additional 0.75 mM MgCl_2_ and 1 μl (10 ng/μL) genomic DNA. The cycling condition consisted of an initial denaturation at 95 °C for 5 min, followed by 25 cycles at 95 °C for 30 s, at 55 °C for 30 s and at 72 °C for 30 s. Successful amplification was verified on agarose gel. The duplicated PCRs were pooled and the PCR products were subsequently purified using AMPure XL magnetic beads (Beckman Coulter, Indianapolis, USA). The purified PCR products were then quantified fluorometrically (Qubit Fluorometer, Thermo Fisher, Waltham, USA) and 50 ng of each sample was pooled prior to sequencing on the Pacific Biosciences sequencing platform with SMRT cell V3 RSII (Pacific Biosciences, Meleno Park, USA) at Uppsala Genome Center, Science for Life Laboratory, Uppsala University, SE-752 37 Uppsala, Sweden.

### 2.5 Bioinformatics and species diversity analysis

The sequencing pools were demultiplexed using Lima 2.7.1 (Pacific Biosciences of California, Inc., available at: https://github.com/PacificBiosciences/barcoding) with the command: lima reads. fq.gz barcodes. fasta demux. fastq –hifi-prefix SYMMETRICS. Amplicon sequence variants (ASVs) were inferred from the sequenced dataset using the DADA2 package in R (Callahan et al., 2016) as outlined on www.nemobiome.ca by Avramenko et al. (2015), with modifications for PacBio error rates. Reads containing unresolved nucleotides (maxN = 0) were removed and primers on both ends were removed from the amplicon sequences using dada2removePrimers. Sequences with a higher-than-expected error number (maxEE = 2) and sequences shorter than 200 bp were also removed. The sequencing error rates were estimated and used to correct the dataset with the functions learnErrors and errorEstimationFunction = PacBioErrfun. The data were then de-replicated using derepFastq and the samples were inferred with the de-replicated dataset as input data (dada). Chimeras were removed using removeBimeraDenovo (method = ‘consensus'). Taxonomic assignment of ASVs was performed with dada2:assignTaxonomy using the taxonomic ITS2 database (v1.4) (Workentine et al., 2020). The database only contains complete sequences, and in order to verify the correct species assignment and fill in unresolved ASVs, these were amended based on identity after using the NCBI BLAST program (Avramenko et al., 2015; Workentine et al., 2020). Species assignment to an ASV was considered if the NCBI BLAST identity was ≥98% similar to the reference sequence. To account for contaminations, singleton reads of each ASV, as well as ASVs with a read count of <0.5% of the total per sample, were filtered out (Baltrusis et al., 2022). Furthermore, samples with less than 200 reads were removed from further analyses (Halvarsson and Höglund, 2021). All ASVs identified to the same species were merged to a single taxa. The final dataset was used for statistical analyses, with all statistical analyses for the bioinformatics conducted using general linear models to infer statistical difference where not stated otherwise. The number of parasite species per individual (species richness) was calculated by summing the species present in each individual (Ludwig and Reynolds, 1988). Alpha indices (Inverse Simpson's diversity index and Shannon-Wiener index (H′)) were calculated using the R package vegan v.2.6–4 using the relative frequencies after standardizing the read counts (Oksanen et al., 2022).

### 2.6 Questionnaire data

All participating establishments were asked to fill in a questionnaire regarding type of establishment (primary function, total number of horses, estimated number of new horses arriving per year), pasture management, anthelmintic treatment routines and management of new arrivals (anthelmintic treatment, quarantine) (Suppl. Table 1). Possible associations between the questionnaire responses and the result of the FECRTs (Table 2) were evaluated using the package xgboost v.1.6.0.1 (Chen and Guestrin, 2016) that implements linear model solver and tree learning algorithms in R v4.2.2 (Team, 2022). To address over-dispersion, factors that were most strongly associated with the outcome of the FECRT were then evaluated using generalized linear models (GLM) with quasipoisson error distribution. The significance was set at the 0.05 level. In a step-wise manner, non-significant factors were removed starting backwards from the factors that showed the least association, until only significant terms remained. The association of age and pre-treatment FECs with the reduction in egg excretion post-treatment was also calculated on an individual horse basis. Correlations between these terms were visualized and significance was calculated with package ggplot2 (Wickham, 2016).

**Table 1:** Selected equestrian establishments, specified by type of establishment and number and age of participating horses, with each establishments’ mean (range) EPG pre- and post-PYR treatment, total number of counted eggs and calculated 90% upper confidence limit (UCL) and 90% lower confidence limit (LCL) of the FECR at two weeks post-treatment. Each establishment is classified as susceptible or resistant in response to PYR treatment as defined by the new WAAVP Guidelines (Kaplan et al., 2023).

Farm no	Type of establishment	Participating horses (total horses at establishment)	Mean age (median, range)	Mean (range) EPG pre-treatment	Mean (range) EPG post-treatment	Total number of eggs counted	UCL and LCL at 90% CI at two weeks post-treatment	Classification (R^a^, S^b^)(Kaplan et al., 2023)
1	livery stable	7 (11–20)	11.3 (8, 4–25)	444 (225–925)	67 (0–188)	504	72.2–94.0%	R
2	livery stable	7 (11–20)	12 (8, 6–32)	365 (175–450)	9 (0–13)	420	95.2–99.2%	S
3	riding school	9 (>40)	12.7 (14, 7–16)	580 (400–900)	11 (0–38)	848	96.8–99.0%	S
4	livery stable, stud farm	8 (11–20)	10.1 (7.5, 2–23)	388 (113–650)	6 (0–25)	420	96.7–99.5%	S
5	riding school	10 (>40)	15.1 (14, 9–26)	542 (375–938)	11 (0–50)	772	95.6–99.4%	S
6	livery stable	6 (31–40)	9.3 (9.5, 4–14)	307 (125–438)	7 (0–25)	320	95.2–99.6%	S
7	livery stable	9 (31–40)	15.9 (17, 5–23)	446 (175–1188)	72 (0–288)	640	65.9–95.5%	R
8	livery stable, riding school	10 (>40)	12,7 (13, 8–19)	478 (163–1113)	28 (0–125)	782	89.5–97.5%	R
9	riding school	9 (31–40)	16.4 (15, 9–23)	589 (250–1138)	90 (0–613)	872	62.9–97.8%	R
10	livery stable	8 (11–20)	11.4 (12, 4–17)	456 (200–888)	65 (0–250)	602	72.6–94.9%	R
11	livery stable	8 (8–10)	7 (4, 2–20)	487 (200–925)	100 (0–313)	640	63.7–91.9%	R
12	livery stable, riding school	6 (21–30)	15.8 (16, 7–24)	559 (100–925)	68 (13–213)	566	76.5–95.7%	R
13	livery stable, stud farm, training/sales stable	10 (>40)	9.1 (7.5, 4–17)	319 (125–763)	6 (0–25)	500	95.8–99.4%	S
14	racing stable	8 (>40)	5.6 (4.5, 2–10)	387 (113–913)	116 (0–738)	524	25.7–96.3%	R
15	livery stable, stud farm	8 (31–40)	5.3 (4, 1–14)	503 (300–1288)	105 (0–425)	770	56.8–94.0%	R
16	livery stable, riding school	9 (21–30)	12.1 (10, 6–23)	368 (125–650)	12 (0–88)	520	91.1–99.5%	S
Overall	N/A	132	11.5 (12, 1–26)	466 (100–1288)	50 (0–613)	N/A	85.6–92.4%	R

a Resistant.

b Susceptible.

**Table 2:** Strategies employed for new arrivals and general anthelmintic treatment routines at the participating establishments.

Question number (from questionnaire data)	Response alternatives	Response (%)
Q3: Average number of new horses/year	>5/year	25%
	2-3/year	56%
	1/year	13%
	None	6%
Q4: Management of new arrivals: anthelmintic treatment of new horses	Yes	63%
	No	13%
	Based on faecal sample	25%
Q4: Management of new arrivals^e^: quarantine of new horses	Separate box/paddock >1 week	31%
	Separate box/paddock ≤1 week	38%
	No quarantine	31%
Q10: Anthelmintic routine^e^	FEC and diagnostics *S. vulgaris* at least once/year	81%
	Anthelmintic treatment only if indicated by faecal sample	63%
	Anthelmintic treatment once per year regardless diagnostics	6%
	Anthelmintic treatment 2–4 times/year regardless diagnostics	0%
Q11: Anthelmintic drug (s) used past two years	ML^a^	19%
	ML^a^, PYR^b^, BZ^c^, ML^a^ + PRZQ^d^	13%
	ML^a^, ML^a^ + PRZQ^d^	6%
	ML^a^, BZ, ML^a^ + PRZQ^d^	0%
	ML^a^, PYR^b^	56%
	ML^a^, PYR^b^, ML^a^ + PRZQ^d^	6%
	ML^a^, PYR^b^, BZ^c^	0%
	ML^a^, BZ^c^	0%

e More than one alternative possible.

a Macrocyclic lactone.

b Pyrantel.

c Bensimidazole.

d Praziquantel.

## 3 Results

### 3.1 Establishments: demographic data and geographical distribution

**Fig. 1:**
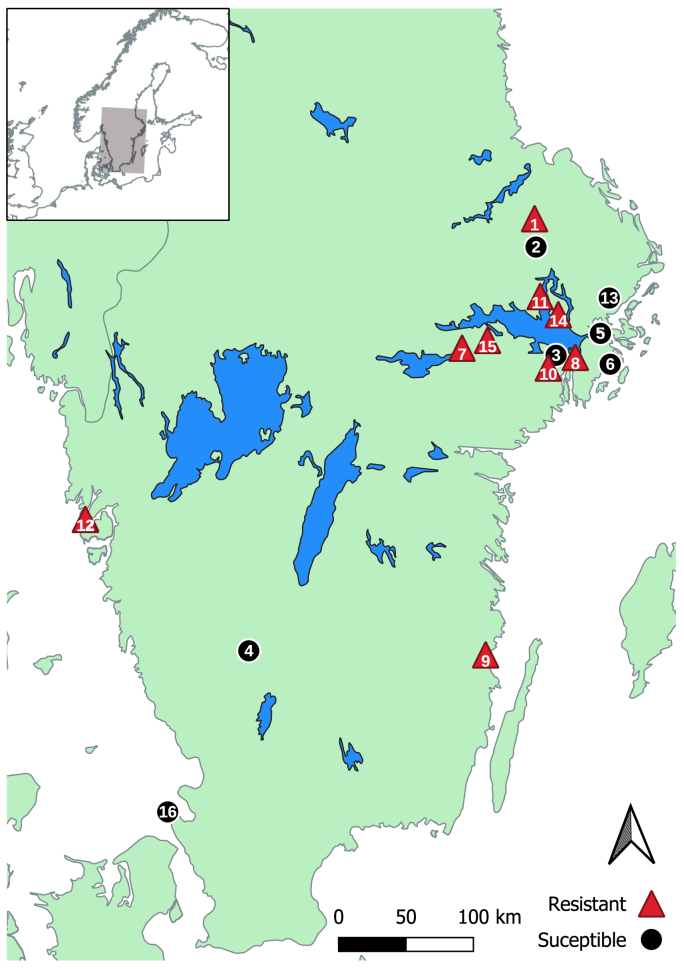
Geographical distribution of the participating equestrian establishments, depicting also their classification as resistant or susceptible.

A total of 16 equestrian establishments with at least six individual horses excreting a minimum 100 EPG prior to anthelmintic treatment were included in the FECRT analyses, giving a total number of participating horses of 132. The types of equestrian establishments, including total number of horses as well as the number of participating horses and their ages (mean, median, range), participating in the study are presented in Table 2. Surprisingly, there was no significant association between pre-treatment FECs and age (R = −0.028; p = 0.75). A variety of breeds were represented: Icelandic horses (31%), pony breeds (27%), warmbloods (26%), Thoroughbreds (8%), cross-breeds (5%), cold bloods (2%) and Standardbred trotters (1%), with one horse of unknown breed. All farms apart from three were located in the south eastern part of Sweden. Two farms (farms 12 and 16) were located in the south western part of Sweden and farm 4, in a more central area of southern Sweden (Fig. 1).

### 3.2 Fecal egg count reduction tests (FECRTs)

The total number of counted eggs for each establishment, the mean and range of the FECs pre- and post-treatment, as well as the results of the FECRTs and subsequent classification according to the current WAAVP-sanctioned guidelines (Kaplan et al., 2023), are shown in Table 1. Out of the 16 farms, seven were classified as susceptible, with nine farms showing resistance to PYR treatment. The establishments’ geographical distribution and FECRT classifications are depicted in Fig. 1. Using the mean pre-treatment FECs for each establishment, power calculations showed that nine farms had fewer than the required number of horses to reach ≥80% power for the outcome: positive evidence of “susceptibility” should the efficacy be adequate (Denwood et al., 2023). However, six of these farms were classified as resistant, and did reach ≥80% power for the outcome: positive evidence of “resistance” should the efficacy be reduced. Thus, in total, the classification of three farms ran the risk of type I error (farms 2, 4 and 6). At an individual level, treatment efficacy was significantly associated with age, with older horses exhibiting a higher FECR at 14 days (R = 0.25; p = 0.004). However, pre-treatment FECs were not associated with efficacy (R = −0.079; p = 0.37).

### 3.3 Questionnaire results

#### 3.3.1 Pasture management

The majority of establishments used separate summer and winter paddocks (63%). However, regular fecal removal in the summer pastures on an at least weekly basis was rare, with only three establishments (19%) declaring to do so. Seven establishments (44%) did not utilize fecal removal during the summer season. Harrowing or topping of the pasture once or twice per year was performed by the majority of respondents during the summer months (56%). Other pasture management methods were rarely employed, with only one establishment utilizing co-grazing with cattle and another responder declaring to rest pastures regularly. There were no statistically significant associations between pasture management methods, type of establishment, total number of residing horses and the establishments’ FECRTs.

#### 3.3.2 Anthelmintic routines

The results of the questionnaire regarding anthelmintic routines and management of new horses are summarized in Table 2. There was a significant association between the FECRT and the establishments’ anthelmintic routine for treatment of new arrivals, with significantly lower anthelmintic efficacy, as demonstrated by a significantly lower FECR after treatment, if new horses were not routinely treated with an anthelmintic drug at arrival (p = 0.019) or treated only as directed by a fecal test result (p = 0.035).

### 3.4 Molecular results

**Fig. 2:**
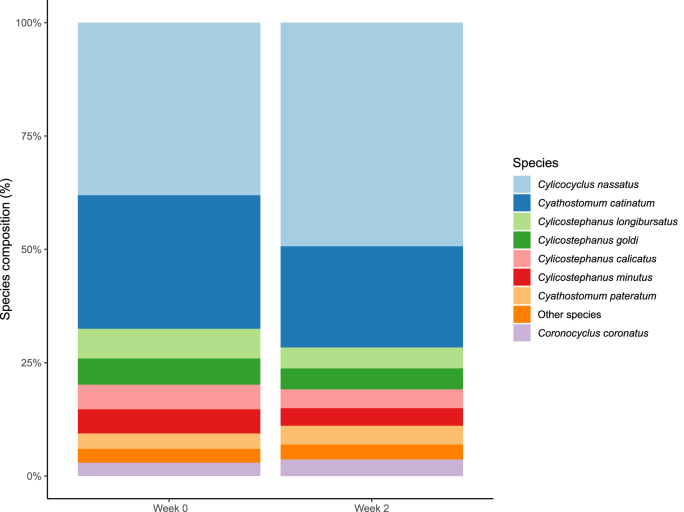
Species composition summary bar plot for week 0 and week 2 showing the mean abundance of the eight most abundant nematode species detected in all fecal samples (week 0: n = 115; week 2: n = 70).

Out of the 264 samples, 49 were excluded due to a yield of 0 EPG at week 2. A further 19 samples (seven samples at week 0 and 12 samples at week 2) were excluded due to laboratory technical issues. Of the remaining 196 samples, 11 samples (10 samples at week 0 and one sample at week 2) did not fulfill the minimum sequencing depth of 50 reads and were removed, resulting in a total of 185 samples for determination of species composition. After quality filtering and ASV clustering, 466,990 reads remained corresponding to a mean of 2524 reads per sample (ranging from 52 to 6652). Subsequent to ASV clustering, the reads were merged to species level based on similarity from NCBI BLAST, resulting in a total of 32 nematode taxa. One of these taxa did not resolve to species level and is named *Cylicostephanus* sp. The occurrence and relative mean abundance of these 32 nematode species are shown in Fig. 2 and Suppl. Table 2.

#### 3.4.1 Species composition and diversity measures prior PYR treatment

**Fig. 3:**
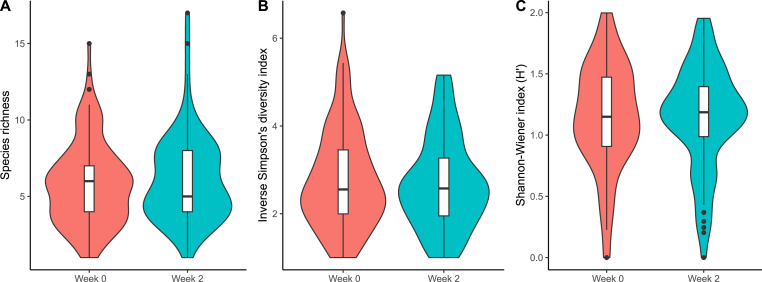
A. Parasite diversity plot showing the median species richness at week 0 and week 2; B. Parasite diversity plot showing the Inverse Simpson's diversity index at week 0 and week 2; C. Parasite diversity plot showing the Shannon-Wiener index H′ at week 0 and week 2. Boxplots inside the violin plots display median values. Sample size is 115 horses on 16 farms at week 0 and 70 horses on 16 farms at week 2.

The average abundance for the eight most common species (*Cyc. nassatus, Cya. catinatum, Cys. longibursatus, Cys. calicatus, Cys. goldi, Cys. minutus, Cor*. *coronatus* and *Cya*. *pateratum*) accounted for 97% of all sequence reads prior to PYR treatment (week 0) (Fig. 2 and Suppl. Table 2). Of these, *Cyc. nassatus* and *Cya. catinatum* had the highest occurrence (95% and 77%, respectively) and also accounted for 68% of all sequence reads. Although the third most common species, *Cys*. *minutus,* had a high occurrence (71%), it had a low mean relative abundance in comparison to *Cyc. nassatus* and *Cya. catinatum,* amounting to approximately 5% of all sequence reads. Similarly, the third most abundant species, *Cys. longibursatus*, had a low mean relative abundance (6.5%) compared with *Cyc. nassatus* and *Cya. catinatum*. In addition to the eight most common species, another 21 species with a low occurrence and a low relative mean abundance were found prior PYR treatment (Suppl. Table 2). The mean and median species richness before PYR treatment was 5.8 and 6.0 species per sample, respectively, with a range of 1–15 species (Fig. 3A). Species richness was significantly associated with FECs, with lower species richness at higher FECs at week 0 (R = - 0.18, p = 0.059). The diversity indices indicated that although *Cyc. nassatus* had the highest abundance, alpha diversity was not dominated by a single species (e.g. Inverse Simpson's diversity index = 1 and Shannon-Wiener index (H′) = 0) (Fig. 3B–C).

#### 3.4.2 Species composition and diversity measures after PYR treatment

The same eight most common species that were found prior to PYR treatment also showed the highest occurrence 14 days after PYR administration (Suppl. Table 2). In addition, there were no major changes in relative mean abundance from week 0 to week 2, with these eight most common species again accounting for 97% of all sequence reads (Fig. 2, Suppl. Table 2). Similar to prior PYR treatment, even if *C. nassatus* had a high abundance, the indices indicated that the diversity after PYR treatment was not dominated by a single species (e.g. Inverse Simpson's diversity index = 1 and Shannon-Wiener index (H′) = 0) (Fig. 3B–C). Of the species with low occurrence and low relative abundance, some differences were, however, seen. For example, *Craterostomum acuticaudatum, Triodontophorus brevicauda* and *Tridentoinfundibulum gobi* were not observed after PYR treatment, whereas *Cylicodontophorus bicoronatus, Cylicostephanus* sp. and *Parapoteriostomum euproctus* were only found after PYR treatment. Furthermore, although still of a low occurrence, in 50% of the more rare species (i.e. species other than the eight most common species), the occurrence was greater post-treatment as compared with prior PYR administration. The mean and median species richness after PYR treatment was 6.2 and 5.0 per individual horse, respectively, with a range of 1–17 species (Fig. 3 A). Despite a reduction in FECs, none of the species diversity measures showed any significant differences between week 0 and week 2 (e.g. species richness (F = 0.74, df = 1, p = 0.39), inverse Simpson's diversity index (F = 0.30, df = 1, p = 0.59) and Shannon-Wiener index (H′) (F = 0.003, df = 1, p = 0.96)) (Fig. 3B–C).

#### 3.4.3 Species composition at farm level

**Fig. 4:**
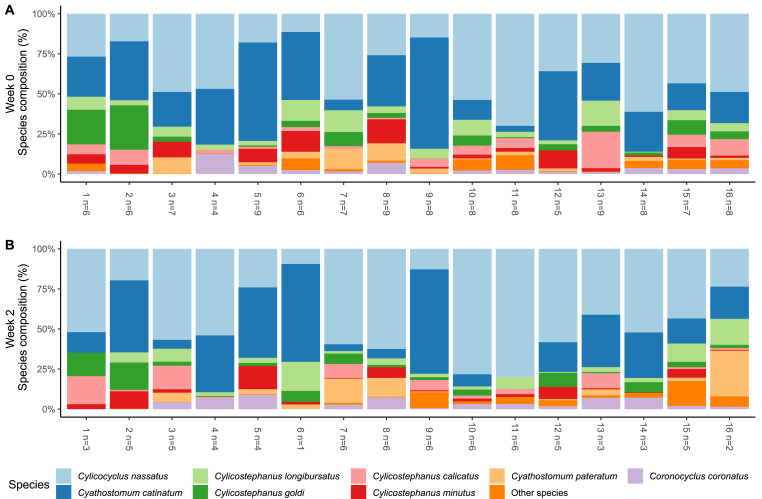
Species composition summary bars for week 0 and week 2 showing the mean abundance of the eight most abundant nematode species detected in fecal samples at each individual participating farm. Note the varying number of horses at week 2 for each farm.

The majority of establishments showed a similar species composition with *Cyc. nassatus, Cya. catinatum, Cys. longibursatus* and *Cys. goldi* amounting to 65–90% of all sequence reads, and with *Cyl. nasstaus* the overall most abundant species both prior to and after PYR treatment (Fig. 4). Due to low number of horses per farm, statistical power was not reached to draw definitive conclusions regarding differences in species diversity between farms. No specific differences in species composition were observed between farms in different geographical areas (Fig. 1).

## 4 Discussion

The present study demonstrated the presence of cyathostomin resistance to PYR treatment in Swedish horse farms, with 9/16 establishments showing AR (UCL <98%). The previously most recent study investigating PYR efficacy in the treatment of cyathostomin infection in Swedish horses was performed in 2007, with one of 22 studied farms deduced to show AR and a further five farms suspected of AR (Osterman Lind et al., 2007). However, different definitions for AR, disparate FECR calculation methods and non-consistent use of repeat testing, together with the inclusion of a low number of horses in the majority of tested farms in the study by Osterman Lind et al. (2007), precludes direct comparisons with the present study. Regardless, the current study confirms the presence of PYR resistance in Sweden, contrasting with ivermectin (IVM), which recently was shown to have retained full efficacy (Hedberg-Alm et al., 2023).

The emergence of cyathostomin resistance to PYR in Sweden is mirrored in the majority of countries where the efficacy of PYR has been evaluated over the past two decades (Nielsen, 2022). However, the results are still somewhat surprising, given the modest use of PYR compared to MLs in Sweden. Official Swedish statistics show that five times as many ML doses were prescribed in 2021 compared with the number of doses of PYR and BZ combined (Girma, 2021). Furthermore, anthelmintic drugs are used conservatively in Sweden, with selective treatment based on fecal diagnostics used by the majority of horse owners and farms treating on a regular basis doing so on average only once per year (Tydén et al., 2019; Hedberg-Alm et al., 2020). Thus, although the overall use of PYR in Swedish horse establishments is low, the results of the present study underline the importance of regular efficacy testing in all horse establishments using the drug. Unfortunately, surveys indicate that the use of FECRTs to determine anthelmintic drug efficacy is seldom employed by horse owners (Elghryani et al., 2019; Dauparaite et al., 2021). Furthermore, many countries have a low grade of veterinary involvement in parasite management (Raś-Noryńska and Sokół, 2017; Elghryani et al., 2019; Dauparaite et al., 2021). National legislation enforcing prescription-only restrictions has been shown to result in notable changes in anthelmintic treatment strategies and a marked increase in adherence to current recommendations, as demonstrated in Sweden, Denmark and the United Kingdom (Nielsen et al., 2014b; Stratford et al., 2014; Tzelos et al., 2019; Hedberg-Alm et al., 2020). Thus, adaptation of regular FECRTs is more likely to occur with increased veterinary participation in parasite control and the imposition of prescription-only restrictions.

In the present study, establishments routinely treating all new arrivals with an anthelmintic drug demonstrated an increased drug efficacy compared with establishments not treating new arrivals or only treating new horses if indicated by fecal testing. This is in agreement with previous data, showing a high level of biosecurity (anthelmintic treatment and quarantine of new horses) to be associated with reduced occurrence of drug resistance (Sallé et al., 2017). However, as was the case in the study by Sallé et al. (2017), a combination of factors are likely to influence the risk of drug resistance. In the present study, data showed considerable overlap between premises as regards to biosecurity measures, with several resistant farms implementing anthelmintic treatment of new horses as a routine measure. Nonetheless, new arrivals pose a risk of introducing resistant parasites (Nielsen et al., 2020).

In total, 31 strongyle species as well as *Trichostrongylus axei* in 14 different genera were found in the current study, corresponding well with a previous study also utilizing fecal cultures for species determination (Poissant et al., 2021). Furthermore, the overall species composition in the present study was in agreement with a large meta-analysis of 37 studies performed over a 25 year period, which also showed *Cyc. nassatus* and *Cya. catinatum* to be the most prevalent (93%) and relatively abundant (20%) Cyathostominae species in equines across seven different geographic regions, together with *Cys. longibursatus,* which was the third most abundant species in the present study (Bellaw and Nielsen, 2020). Similarly, a previous study using DNA sequencing from fecal nematode eggs, demonstrated these same three species to have a 100% occurrence (Mitchell et al., 2019).

Our results can also be compared with a larger study, where they found 23 strongyle species in DNA extracted from 159 pooled fecal samples from 1377 horses (Abbas et al., 2023). Differences in source material for DNA extraction, sequencing platform and cut-offs in the bioinformatics pipelines might affect the discovery of rare species. Although the PacBio sequencing yielded fewer sequence reads compared with Illumina MiSeq (Poissant et al., 2021; Abbas et al., 2023) and differed in the bioinformatics processing, we found a similar number of taxa across the samples. However, it cannot be ruled out that the discovery rate of rare strongyle species is lower due to the lower number of sequence reads from the PacBio platform.

Despite similarities in methodology and overall species detection, the present study showed a surprisingly low mean species richness of only six species per sample (range 1–15), compared with the study by Poissant et al. (2021), where the overall number of species in individual fecal samples in three different geographical regions ranged from 9 to 26 species. All horses in the study by Poissant et al. (2021) were feral or semi-feral with no recent history of anthelmintic exposure, which could possibly serve as an explanation for the larger species richness observed, given that older horses, with a longer history of administered anthelmintic treatments, have been shown to have a lower species richness compared with younger individuals (Kuzmina et al., 2016). However, the species richness demonstrated in the current study is in good agreement with a recent publication utilizing metabarcoding on larval cultures from a small herd of six anthelmintic-naïve Scottish ponies, where species richness was shown to differ significantly both between individual horses as well as with season (Sargison et al., 2022). Thus, individual, geographical, environmental and seasonal variations may influence species diversity and richness (Bellaw and Nielsen, 2020; Poissant et al., 2021; Sargison et al., 2022). Due to the low number of horses per farm, a possible effect of geographical variation on species richness or composition could not be determined in the current study.

In the present study, no substantial change in cyathostomin species composition, richness or dominance occurred as a consequence of PYR treatment, with *Cyc. nassatus* and *Cya. catinatum* dominating egg production both prior to and after anthelmintic treatment. The lack of effect on species composition and diversity indices after PYR treatment agrees with a previous study showing no significant effect on overall Cyathostominae diversity at 42-days post PYR treatment, apart from a significant reduction in *C. longibursatus* abundance (Boisseau et al., 2022). An earlier publication, using different methodology, instead showed an increase in the relative abundance of *Cys. longibursatus* after PYR treatment, which was speculated to be due to a shorter pre-patent period in this species, and not as a result of AR (Kooyman et al., 2016). Nonetheless, the results of the present study, given that the majority of farms showed AR, appear to suggest that PYR resistance occurred across all of the species. This is a surprising finding and the notion of single species developing AR, as has been shown in beef calves, was not supported by the present results (Avramenko et al., 2017). Furthermore, our results contrast with findings related to IVM resistance in horses, where one species, *Cyc. insigne*, was clearly over-represented when luminal fourth-stage larvae were counted two weeks after IVM treatment (Nielsen et al., 2022). In addition, Nielsen et al. (2022) showed *Cyc. nassatus* to be the main contributor to early egg production after moxdectin treatment.

In conclusion, the present study confirmed the presence of PYR resistance in Sweden, despite the high level of selective anthelmintic treatment regimens used in the studied equine population and thus an overall low use of the drug. Moreover, no significant effect of PYR treatment on cyathostomin species composition or species diversity was demonstrated. Although the drug may still be a viable option on some farms, the results highlight the dynamic nature and emergence of anthelmintic resistance with time, and hence the importance of implementing regular anthelmintic efficacy testing, regardless the intensity of anthelmintic drug use.

## Funding

This work was supported by the Foundation for Swedish and Norwegian Equine Research, grant number H-15-47-097 and by P O Lundell's foundation.

## Institutional review board statement

Ethical review and approval were waived for this study in accordance with relevant guidelines and regulations issued by the Swedish Board of Agriculture's regulations and general advice on laboratory animals (SJVFS, 2019:9, case no. L150).

## Availability of data and materials

Questionnaire responses, raw metabarcoding sequence data and sample information are available in the BioStudies database (https://www.ebi.ac.uk/biostudies/) under accession number S-BSST1119.

## Declaration of competing interest

The authors declare the following financial interests/personal relationships which may be considered as potential competing interests: Eva Tyden reports financial support was provided by The Swedish-Norwegian Foundation for Equine Research.
