# Supplementary material for: Demonstration of reduced efficacy against cyathostomins without change in species composition after pyrantel embonate treatment in Swedish equine establishments
Source: Int J Parasitol Drugs Drug Resist. 2023 Nov 14;23:78–86. doi: 10.1016/j.ijpddr.2023.11.003 (PMC10690405; doi:10.1016/j.ijpddr.2023.11.003)
Supplement: Multimedia component 1 [file mmc1.docx]

Suppl. Table 1. Questionnaire data collected from participating establishments.

| Information | Descriptor |
| --- | --- |
| Q1: Number of horses | i) 8-10 ii) 11-20 iii) 21-30 iv) 31-40 v) > 40 |
| Q2: Type of establishment* | i) livery ii) racing iii) stud iv) riding school v) other |
| Q3: Average number of new horses/year | i) None ii) one/year iii) 2-3/year iv) > 5/year |
| Q4: Management of new arrivals* | i) Anthelmintic treatment ii) Anthelmintic treatment after faecal sample  iii) no treatment iv) separate box/paddock > 1 week v) separate box/paddock ≤ 1 week vi) no separate box/paddock |
| Q5: Separate summer/winter paddock | i) yes ii) no |
| Q6: Faecal removal winter | i) several times/week ii) once/week iii) once/month iv) once/6 months v) once/year vi) never |
| Q7: Faecal removal summer | i) several times/week ii) once/week iii) once/month iv) once/6 months v) once/year vi) never |
| Q8: Harrowing or topping of pasture* | i) summer: once/year ii) summer: twice/year iii) summer: > twice/year  iv) winter: once/year v) winter: twice/year vi) winter: > twice/year  vii) never |
| Q9: Other pasture management | Free text |
| Q10: Anthelmintic routine* | i) treatment only if indicated by faecal sample  ii) use of FECs and diagnostics for *S. vulgaris* at least once/year  iii) routine deworming once/year iv) routine deworming 2–4 times/year |
| Q11: Anthelmintic drug (s) used past two years* | i) FBZ^a^ ii) PYR^b^ iii) ML^c^ iv) ML + PRZQ^d^ v) no recollection vi) other |

* More than one alternative possible. ^a^ fenbendazole, ^b^ pyrantel, ^c^ macrocyclic lactone, ^d^ macrocyclic lactone and praziquantel combination
