# Supplementary material for: Demonstration of reduced efficacy against cyathostomins without change in species composition after pyrantel embonate treatment in Swedish equine establishments
Source: Int J Parasitol Drugs Drug Resist. 2023 Nov 14;23:78–86. doi: 10.1016/j.ijpddr.2023.11.003 (PMC10690405; doi:10.1016/j.ijpddr.2023.11.003)
Supplement: Multimedia component 2 [file mmc2.docx]

Suppl. Table 2. Nematode species found in faecal samples prior PYR treatment (week 0) and at two weeks post-treatment (week 2) with their respective occurrences expressed as the percentage of individuals

infected out of the total number of individuals sampled and as their respective mean abundances expressed as a percentage of the total number of sequence reads.

|  | **Week 0 (n = 115)** | | | **Week 2 (n = 70)** | | | **Genbank^®^ ID** |
| --- | --- | --- | --- | --- | --- | --- | --- |
| **Species** | **Individuals** | **Occurrence (%)** | **Mean abundance (%)** | **Individuals** | **Occurrence (%)** | **Mean abundance (%)** |  |
| ***Cylicocyclus nassatus*** | **109** | **95** | **38.0** | **70** | **100** | **49.3** | **MT193649.1** |
| ***Cyathostomum catinatum*** | **89** | **77** | **29.5** | **49** | **70** | **22.3** | **MT193653.1, KY495603.1** |
| ***Cylicostephanus longibursatus*** | **70** | **61** | **6.5** | **40** | **57** | **4.6** | **MW282923.1, MW282936.1, MW282925.1** |
| ***Cylicostephanus calicatus*** | **46** | **40** | **5.5** | **24** | **34** | **4.2** | **MW367018.1, MW367019.1, MW367020.1** |
| ***Cylicostephanus goldi*** | **58** | **50** | **5.8** | **39** | **56** | **4.6** | **KM085357.1** |
| ***Cylicostephanus minutus*** | **82** | **71** | **5.3** | **53** | **76** | **3.9** | **MZ435501.1, MW282952.1, MW282946.1, AJ004843.1** |
| ***Coronocyclus coronatus*** | **51** | **44** | **2.9** | **42** | **60** | **3.7** | **KY747448.2** |
| ***Cyathostomum pateratum*** | **38** | **33** | **3.4** | **31** | **44** | **4.2** | **KF850628.1** |
| *Cylicocyclus ashworthi* | 17 | 15 | 1.0 | 15 | 21 | 1.5 | MW198060.1 |
| *Cylicocyclus leptostomus* | 12 | 10 | 0.5 | 8 | 11 | 0.7 | KP693432.1 |
| *Cylicocyclus insigne* | 13 | 11 | 0.5 | 9 | 13 | 0.2 | Y08588.1, JQ906419.1 |
| *Coronocyclus labiatus* | 13 | 11 | <0.1 | 3 | 4 | <0.1 | KY747447.2, MW243589.1 |
| *Coronocyclus labratus* | 2 | 2 | <0.1 | 2 | 3 | <0.1 | KY747449.2, JN786949.2 |
| *Petrovinema poculatum* | 5 | 4 | <0.1 | 6 | 9 | 0.3 | KP693443.1 |
| *Cyathostomum montgomeryi* | 6 | 5 | 0.1 | 2 | 3 | <0.1 | MZ435572.1 |
| *Strongylus edentatus* | 5 | 4 | 0.3 | 5 | 7 | 0.1 | MT193648.1 |
| *Parapoteriostomum mettami* | 9 | 8 | <0.1 | 3 | 4 | <0.1 | KP693435.1 |
| *Cylicostephanus bidentatus* | 8 | 7 | <0.1 | 7 | 10 | 0.1 | AJ004839.1 |
| *Oesophagodontus robustus* | 6 | 5 | <0.1 | 1 | 1 | <0.1 | Y08592.1 |
| *Craterostomum acuticaudatum* | 4 | 3 | <0.1 | 0 | 0 | 0 | AJ228236.1 |
| *Cylicocyclus ultrajectinus* | 10 | 9 | <0.1 | 4 | 6 | <0.1 | KP693431.1 |
| *Trichostrongylus axei* | 3 | 3 | <0.1 | 4 | 6 | 0.2 | ON677951.1 |
| *Strongylus vulgaris* | 2 | 2 | <0.1 | 3 | 4 | <0.1 | OP672317.1 |
| *Gyalocephalus capitatus* | 3 | 3 | <0.1 | 2 | 3 | <0.1 | KP693442.1 |
| *Poteriostomum imparidentatum* | 2 | 2 | <0.1 | 1 | 1 | <0.1 | KY495604.1 |
| *Triodontophorus serratus* | 1 | 1 | <0.1 | 4 | 6 | <0.1 | KR296738.1 |
| *Cylicocyclus brevicapsulatus* | 1 | 1 | <0.1 | 1 | 1 | <0.1 | AJ004835.1 |
| *Triodontophorus brevicauda* | 2 | 2 | <0.1 | 0 | 0 | 0 | MT193652.1 |
| *Cylicodontophorus bicoronatus* | 0 | 0 | 0 | 1 | 1 | <0.1 | MT193662.1 |
| *Tridentoinfundibulum gobi* | 2 | 2 | <0.1 | 0 | 0 | 0 | AF263497.1, AJ004847.1 |
| *Cylicostephanus* sp. | 0 | 0 | 0 | 2 | 3 | <0.1 | MH483944.1, MW282937.1 |
| *Parapoteriostomum euproctus* | 0 | 0 | 0 | 1 | 1 | <0.1 | KP693692.1 |
